# Correlation between serum S100β protein levels and cognitive dysfunction in patients with cerebral small vessel disease: a case–control study

**DOI:** 10.1042/BSR20160446

**Published:** 2017-04-10

**Authors:** Fei Wang, Zhi-Rong Zou, Dong Yuan, Yi Gong, Li Zhang, Xun Chen, Tao Sun, Hua-Lin Yu

**Affiliations:** 1 Second Department of Neurosurgery, The First Affiliated Hospital of Kunming Medical University, Kunming 650032, P.R. China; 2 Faculty of Basic Medical Sciences, Kunming Medical University, Kunming 650500, P.R. China

**Keywords:** Cerebral small vessel disease, Cognitive dysfunction, Mini-mental state examination, Montreal cognitive assessment, Serum S100β

## Abstract

The present study was designed to explore the correlation between serum S100β levels and cognitive dysfunction in patients with cerebral small vessel disease (SVD). A total of 172 SVD patients participated in the study, and they were assigned to patients with no cognitive impairment (NCI group) and those with vascular cognitive impairment no dementia (VCIND group). In total, 105 people were recruited into the normal control group. Serum S100β protein level was detected by ELISA. A receiver operating characteristic (ROC) curve was employed for the predictive value of serum S100β in diagnosing SVD with cognitive dysfunction. Pearson correlation analysis was used to examine the association of S100β level with mini-mental state examination (MMSE) and Montreal cognitive assessment (MoCA) and the association of S100β levels with hypertension. Logistic regression analysis was used to analyze risk factors of SVD. The serum S100β levels in the VCIND group were higher than those in the NCI and normal control groups. Logistic regression analysis revealed that a high serum S100β protein level, hypertension, and high low density lipoprotein-cholesterol (LDL-C) level were the independent risk factors for SVD. In addition, hypertension patients showed higher S100β levels than those with normal blood pressure and the normal control group, and there was a positive correlation between S100β level and blood pressure. The concentration of serum S100β level was related to impairment of cognition function of VCIND patients, therefore, early detection of serum S100β was of great value for diagnosis of SVD.

## Introduction

Cerebral small vessel disease (SVD) describes a series of pathological processes caused by multiple etiologies that produce great effects on the small arteries, venules, arterioles, and capillaries of the brain, which is considered a primary cause of cognitive decline and function loss in elderly patients and plays a key role in cerebrovascular diseases [1]. Brain parenchymal arterioles play a crucial part in vascular resistance [2] and are important for the maintenance of normal blood flow to structures in the subsurface brain [3]. The human brain needs a disproportionate amount of energy generated by the body [4]. To transmit the energy efficiently and protect the brain from ischemic damage and hypoperfusion, the cerebral vasculature is equipped with developed mechanisms that maintain constant cerebral blood flow when the arterial pressure fluctuates and satisfies nutrient demands when local brain activity increases [5]. However, SVD can chronically and significantly prevent cerebral vasculature from meeting these requirements owing to various functional and structural changes that eventually lead to cognitive decline in elderly patients [6–8]. As cognitive impairment has been shown to be associated with S100 protein in several diseases including mild traumatic brain injury [9], chronic cerebral hypoperfusion [10], and Parkinson’s [11], we reasonably hypothesize that the S100β protein is associated with cognition impairment in SVD patients.

The S100 protein family belongs to a group of acidic proteins that can connect with calcium and affect various cellular responses in the calcium signaling transduction pathway [12]. One member of the S100 protein family in the central nervous system is of great importance i.e. S100β, a small Ca^2+^-binding acidic protein. This protein is abundant within the central nervous system, is secreted by oligodendrocytes as well as astrocytes, and is also expressed in neurons and ependyma [13]. S100β is involved in the regulation of cell shape, cell growth, energy metabolism, cell-to-cell communication, contraction, and intracellular signal transduction [14]. As a small protein with a weight of 21 kDa in a homo-dimeric form, S100β can pass through the blood-brain barrier; thus, this protein is detectable in peripheral blood as a brain-derived protein [15]. S100β measurement has been reported to significantly reflect the S100β concentration in healthy individuals as well as in patients with various neurological dysfunctions [13]. Additionally, elevated S100β levels are related to mood disorders, hypoperfusion, and the poor clinical outcome of intracerebral hemorrhage [14,16,17]. However, a previous study only measured the difference in S100β levels between SVD patients and healthy outpatients by ELISA [18]. Therefore, our study aimed to investigate the possible correlation between the serum S100β level and cognitive dysfunction in SVD before and after surgery based on the mini-mental state examination (MMSE) and Montreal cognitive assessment (MoCA).

## Materials and methods

### Study subjects

One hundred and seventy-two patients, who received treatment at the First Affiliated Hospital of Kunming Medical University between January 2011 and March 2014 were recruited. All SVD patients were confirmed by imageology under the following criteria: (i) leukoaraiosis, lacunar infarction, cerebral microbleed or enlarged vascular spaces present in the image; (ii) no subcortical and watershed infarction; and (iii) no evident narrow area observed in the carotid artery. All patients were assigned to the vascular cognitive impairment no dementia (VCIND) group (56 males and 36 females) and the no cognitive impairment (NCI) group (58 males and 22 females). The patients were included in the NCI group if they had no cognitive decline, their overall cognitive level was normal and they reached a score greater than 26 according to MoCA [19]. Patients were included in the VCIND group if they had cognitive decline, their MoCA score was less than 26, and their cognitive impairment failed to reach the clinical dementia standard according to the Diagnostic and Statistical Manual (revision IV) [20]. Exclusion criteria: (i) patients who had intracranial tumors, a closed brain injury, multiple sclerosis, lacunar infarction, or other central nervous system diseases; (ii) patients who were addicted to alcohol, narcotics, and other psychiatric drugs; (iii) patients who had severe dysfunction in the heart, liver, kidney, hemopoietic system, and thyroid; (iv) patients who took medication with vitamin preparation and nootropics before diagnosis; (v) patients who had evident visual and auditory disturbance or failed to complete related neuropsychological tests; (vi) patients who were recognized as recognition impairment, with an AD8 score of more than 2 or the total score of dementia caregivers questionnaire over 56; (vii) patients who suffered from cognition impairment due to other diseases, including tumors, infection, intoxication, metabolic disease (such as insufficiency of vitamin B12 and folic acid intakes), and congenital dysgnosia; (viii) patients who passed the Hamilton depression rating scale (HAMD) and Hachinski ischemic score with exception of pseudocognitive impairment and dementia caused by depression, anxiety, or Alzheimer’s disease. Additionally, 105 healthy subjects (73 males and 32 females) during the same period were enrolled in the normal control group. No significant difference was found between the two groups in terms of age, gender, educational background, or chronic diseases. The study was approved by the Ethics Committee of the First Affiliated Hospital of Kunming Medical University, and all of the subjects signed informed consent.

### Assessment of neurocognitive function

All subjects underwent a neuropsychological test that included the MMSE and MoCA for neurocognitive assessment.

### ELISA

On the second day after clinical evaluation, 5 ml of venous blood was obtained from each subject when they were fasting, and the blood was placed into polyvinyl chloride (PVC) tubes for coagulation. Next, the blood samples were stored in a refrigerator at 4°C for 2 h and were centrifuged at 3000 rpm for 10 min for the separation of blood serum. The supernatants were stored in PVC tubes, which were placed in a −20°C refrigerator. The samples were dissolved at room temperature before detection. The concentration of serum S100β was assessed using a human serum S100β ELISA kit (Roche Diagnostics Corp., Basel, Switzerland). There was a blank well (with no sample, biotin-labeled anti-IgG antibody or streptavidin-horseradish peroxidase (HRP), standard sample well and a to-be-tested sample well. The standard sample, 50 μl, was added into the standard sample well, and 10 μl of the to-be-tested sample was added into the to-be-tested sample well, followed by the addition of 40 μl diluent to reach the ratio of 1:5, sealing, mixing, and incubation at 37°C for 45 min. After samples were diluted using distilled water at the ratio of 1:20, the liquid was removed, and a cleaning solution was then added into each well and allowed to stand for 30 s; the liquid was then aspirated again. The whole washing process was repeated four times. Fifty microliters of anti-IgG antibody was added into each standard sample well and to-be-tested sample well. After reaction at 37°C for 30 min, the wells were washed again. Next, 50 μl of streptavidin-HRP was added into each standard sample well and to-be-tested sample well to react with the samples at 37°C for 30 min, and then the wells were washed. Finally, 50 μl of chromogenic agent A and 50 μl of chromogenic agent B were added into each well to develop the samples at 37°C for 15 min without light. Subsequently, 50 μl of stopping solution was added into each well to terminate the reaction. Within 15 min of termination, the optical density (OD) of each well was measured at a wavelength of 450 nm with the blank well as the zero standard. The blood biochemical index was simultaneously detected using the BS-320 full-automatic biochemical analyzer (Shenzhen Mindary Bio Medical Electronic Co., Ltd, Shenzhen, China) combined with a kit (Nanjing Jiancheng Bioengineering Institute, Jiangsu, China) to detect fasting blood glucose, total cholesterol (TC), triglyceride (TG), high density lipoprotein-cholesterol (HDL-C), and low density lipoprotein-cholesterol (LDL-C) levels. The operation was performed strictly according to the manufacturer’s instructions.

### Statistical analysis

Statistical analysis was conducted with the Statistical Package for the Social Sciences (SPSS) version 21.0 (SPSS Inc.; Chicago, IL, U.S.A.). Continuous data were reported as the mean ± S.D. ($\bar{x}$ ± s). The differences among multiple groups were analyzed by one-way ANOVA. Additionally, the differences between the groups were compared using the Scheffe method. A receiver operating characteristic (ROC) curve was employed for the predictive value of serum S100β in diagnosing SVD with cognitive dysfunction. Pearson analysis was applied for the correlation test, and logistic regression analysis was applied for disease risk factors. *P*<0.05 was regarded as statistically significant.

## Results

### Comparison of clinical characteristics among the NCI group, the VCIND group and the normal control group

After comparison of the NCI and VCIND groups and the normal control group in terms of their age, gender, smoking, alcohol consumption, blood pressure, blood glucose, family medical history, and blood biochemical indexes, it was found that the NCI and VCIND groups exhibited more smoking, higher blood pressure, TC, and LDL-C than the normal control group (all *P*<0.05), while the NCI and VCIND groups and the normal control group had no significant difference in other indicators (all *P*>0.05) (Table 1).

**Table 1:** Comparison of clinical characteristics among the normal control group, the NCI group, and the VCIND group

Clinical characteristic	NCI group	VCIND group	Normal control group	*P*
	(*n*=80)	(*n*=92)	(*n*=105)	
Age (year)	58 (72.50)	56 (60.87)	73 (69.52)	0.229
Male (%)	10.03 ± 1.93	10.17 ± 2.26	10.40 ± 2.32	0.509
Education duration (year)	26 (32.50)	34 (36.96)	21 (20.00)	0.035
Smoking (%)	17 (21.25)	19 (20.65)	25 (23.81)	0.851
Drinking (%)	23.82 ± 2.17	26.39 ± 2.03	19.23 ± 2.03	<0.001
Blood pressure (kPa)	5.50 ± 0.49	5.53 ± 0.53	5.41 ± 0.49	0.22
Blood glucose (mmol/l)	5 (6.25)	7 (7.61)	10 (9.52)	0.709
Cardiovascular disease (%)	5.76 ± 0.66	5.83 ± 0.63	4.81 ± 0.53	<0.001
TC (mmol/l)	1.51 ± 0.20	1.51 ± 0.24	1.49 ± 0.18	0.739
TG (mmol/l)	1.23 ± 0.30	1.25 ± 0.36	1.17 ± 0.30	0.192
HDL-C (mmol/l)	2.66 ± 0.24	2.81 ± 0.35	2.32 ± 0.26	<0.001

### Comparison of neurocognitive function scores among the NCI group, the VCIND group, and the normal control group

The MMSE and MoCA assessments were performed on patients after hospital admission. The data revealed that the MMSE and MoCA scores were decreased in the VCIND group compared with the NCI and normal control groups (all *P*<0.05). The MMSE subitems (recall, executive function, orientation, and calculation) and MoCA subitems (attention, calculation, abstract, visual space, and executive function) were decreased in the VCIND group (*P*<0.05), but no significance was found in the other subitems (*P*>0.05). The MMSE and MoCA in the NCI group were not significantly different from those in the normal control group (all *P*>0.05, Table 2).

**Table 2:** Comparison of MMSE and MoCA scores among the VCIND group, the NCI group, and the normal control group ($\bar{\text{x}}$ ± s)

		VCIND group	NCI group	Normal control group
MMSE	Total score	23.88 ± 3.26	27.34 ± 2.64	27.34 ± 2.47
Subitems	Language/1	0.93 ± 0.25	0.95 ± 0.22	0.92 ± 0.27
	Recall/3	1.79 ± 0.41^▲*^	2.48 ± 0.50	2.55 ± 0.50
	Executive function/5	2.65 ± 0.78^▲*^	3.89 ± 0.42	3.99 ± 0.10
	Orientation/10	8.96 ± 0.55^▲*^	9.56 ± 0.50	9.55 ± 0.50
	Calculation/5	3.59 ± 0.83^▲*^	4.43 ± 0.50	4.39 ± 0.49
	Naming/2	1.92 ± 0.27	1.95 ± 0.22	1.89 ± 0.40
	Repetition/4	4.03 ± 0.18	4.09 ± 0.28	4.05 ± 0.21
MoCA	Total score	18.99 ± 4.39	26.13 ± 2.94	26.20 ± 2.78
Subitems	Delay memory/5	2.84 ± 0.48	3.01 ± 0.49	3.01 ± 0.43
	Language/3	2.05 ± 0.31	2.20 ± 0.40	2.22 ± 0.42
	Attention and calculation/6	2.75 ± 0.98^▲*^	5.65 ± 0.53	5.66 ± 0.52
	Orientation/6	5.45 ± 0.58	5.61 ± 0.49	5.60 ± 0.49
	Visual space and executive function/5	2.02 ± 0.76^▲*^	4.84 ± 0.37	4.87 ± 0.34
	Naming/3	2.90 ± 0.96	2.89 ± 0.39	2.89 ± 0.40
	Abstract/2	0.98 ± 0.33^▲*^	1.93 ± 0.27	1.96 ± 0.19

*, *P*<0.05 when compared with the NCI group; ▲, *P*<0.05 when compared with the normal control group.

### Comparison of the serum S100β protein level among the NCI group, the VCIND group, and the normal control group

**Figure 1:**
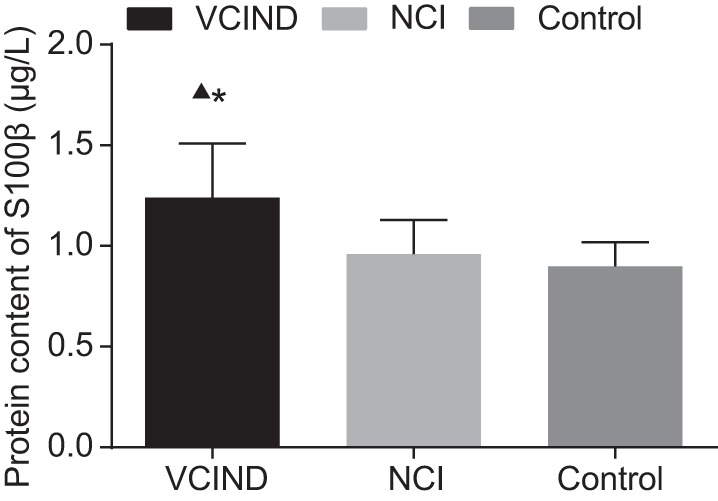
Comparison of serum S100β protein level among the normal control group, the NCI group, and the VCIND group ($\bar{x}$ ± s, µg/l) *, compared with the normal control group, *P*<0.05; ▲, compared with the NCI group, *P*<0.05.

Serum S100β was detected in all subjects on the second day after the assessment and it was found that the serum S100β level in the VCIND group was evidently higher than that in the NCI and the normal control groups (*P*<0.05). Serum S100β in the NCI group was slightly higher than that in the normal control group but showed no significant difference (*P*>0.05, Figure 1).

### ROC analysis of the predictive value of the S100β level in diagnosing SVD with cognitive dysfunction

**Figure 2:**
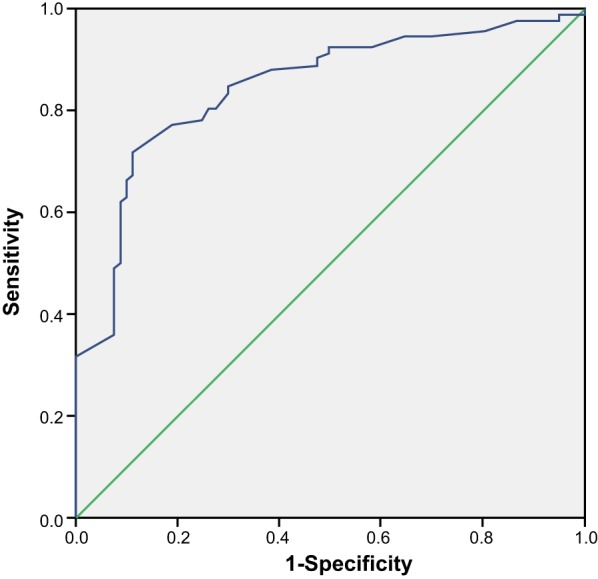
ROC curve analysis of the predictive value of S100β in diagnosing SVD with cognitive dysfunction

The ROC curve (Figure 2) presented the predictive value of the S100β level in diagnosing SVD with cognitive dysfunction, in which the area under curve (AUC) was 0.845, 95% confidence interval (CI) was 0.786–0.905, the sensitivity was 71.74%, specificity was 88.75%, diagnosis threshold was 1.10, positive predictive value was 88.00%, and negative predictive value was 88.00%.

### Correlation analysis between the serum S100β level and neuropsychological assessment in the VCIND group

Serum S100β of patients in the VCIND group was numbered and contrasted with the changes of MMSE and MoCA scores, and also, the Pearson correlation coefficient was adapted to analyze the correlation of serum S100β and neuropsychological score. The results indicated that serum S100β was negatively related to MMSE total scores and its subitems (executive function, orientation, and calculation) (*P*<0.05). Serum S100β was not correlated to language, recall, naming, and repetition (*P*>0.05). Additionally, serum S100β in the VCIND group was negatively associated with MoCA total scores and its subitems (delay memory, language, visual space, executive function, and naming) (*P*<0.05), but it showed no connection with other subitems (attention, orientation, and abstract) (*P*>0.05, Table 3).

**Table 3:** Correlation analysis of serum S100β level with the MMSE and MoCA scores in VCIND patient

	Category	R value	*P*
MMSE	Total grade	–0.62	<0.001
Subitems	Language	−0.114	0.278
	Recall	0.028	0.79
	Executive function	−0.478	<0.001
	Orientation	−0.491	<0.001
	Calculation	−0.466	<0.001
	Naming	−0.063	0.554
	Repetition	−0.201	0.055
MoCA	Total grade	−0.56	<0.001
Subitems	Delay memory	−0.279	0.007
	Language	−0.333	<0.001
	Attention	−0.077	0.464
	Orientation	−0.034	0.744
	Visual space and executive function	−0.516	<0.001
	Naming	−0.276	0.008
	Abstract	0.124	0.241

### Logistic regression analysis for risk factors of cerebral SVD patients

Logistic regression analysis was conducted with the baseline characteristics in which significant difference was demonstrated as independent variables (including smoking, blood pressure, TC, LDL-C levels, and serum S100β), and with whether SVD patients had cognitive dysfunction patients as dependent variables. It revealed that hypertension (odds ratio (OR) =1.440, 95% CI =1.162–1.785), higher LDL-C levels (OR =6.510, 95% CI =1.229–34.487), and higher serum S100β (OR =228.707, 95% CI =33.590–1557.233) were independent risk factors for SVD (all *P*<0.05, Table 4).

**Table 4:** Logistic regression analysis for risk factors of cerebral SVD patients

	B	S.E.M.	Wald	*P*	OR (95% CI)
Smoking	0.076	0.406	0.035	0.852	1.079 (0.487–2.390)
Blood pressure	0.247	0.123	4.041	0.044	1.281 (1.006–1.630)
TC	0.146	0.306	0.226	0.635	1.157 (0.635–2.108)
LDL-C	1.704	0.74	5.299	0.021	5.498 (1.288–23.470)
Serum S100β	4.254	1.326	10.29	0.001	70.382 (5.232–946.759)

B, partial regression coefficient.

### Comparison of S100β levels between patients with hypertension and those with normal blood pressure

**Figure 3:**
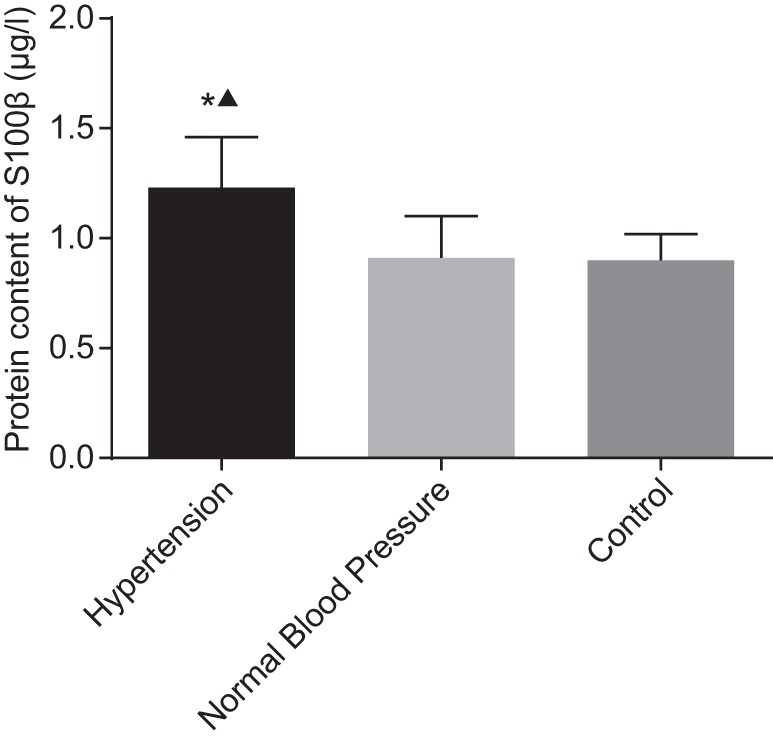
Comparison of serum S100β level among the hypertension group, the normal blood pressure group, and the control group *, compared with the normal blood pressure group, *P*<0.05; ▲ compared with the control group, *P*<0.05.

**Figure 4:**
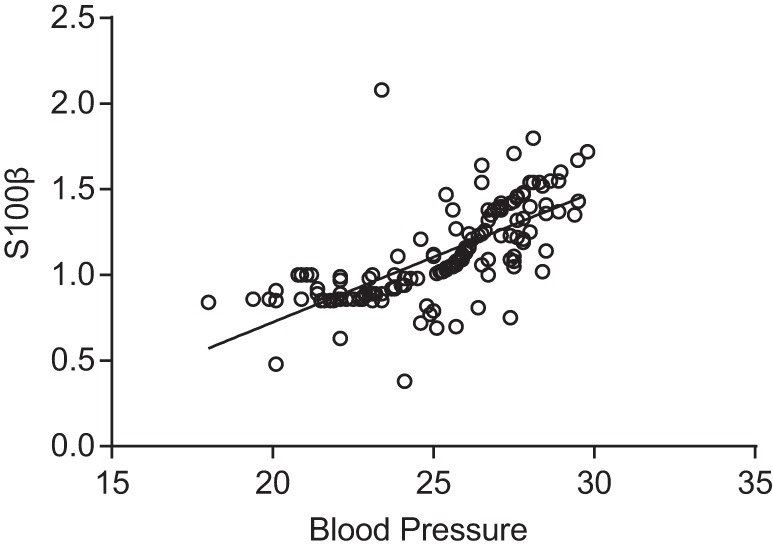
Correlation between SVD patients’ blood pressure and S100β level after Pearson regression analysis

Patients were divided into normal blood pressure group (*n*=62) and hypertension group (*n*=100) based on their blood pressure, and their serum S100β level was compared and analyzed. The results revealed that patients in the hypertension group exhibited a significantly higher serum S100β level than those with normal blood pressure (*P*<0.05), whereas no statistical differences were found between patients in the normal blood pressure group and those in the normal control group (*P*>0.05) (Figure 3). The result of Pearson regression analysis showed S100β was positively correlated to blood pressure (r =0.706, *P*<0.05) (Figure 4).

## Discussion

The results of the present study show that serum S100β levels are increased in VCIND patients. Additionally, concerning MMSE and MoCA, VCIND patients acquired lower grades than those in the NCI group and the normal control group, and the MMSE recall and executive function subitems as well as the MoCA attention, delay memory abstract, visual space, and executive function subitems are decreased in patients with VCIND compared with those in the NCI and normal control groups. Regarding the correlation between serum S100β levels and MMSE and MoCA grades, our findings reveal that S100β levels exhibit a negative correlation with the total grade and the subitems of executive function, orientation, and calculation in MMSE, and the total grade and subitems of delay memory, attention, visual space, executive function, and naming in MoCA. Collectively, these data suggest that an elevated plasma S100β level is an independent risk factor for cognitive dysfunction in SVD. This finding is associated with the degree of cognitive impairment of VCIND patients, and the early assessment of the plasma S100β protein level is significant for the diagnosis of SVD patients with VCIND.

Importantly, we find that during general anesthesia, the S100β protein level is increased in the VCIND group compared with that in the NCI group and normal control group. Increased serum S100β levels are implicated in various neurological dysfunctions, such as mood disorders, hypoperfusion, poor clinical effects of intracerebral hemorrhage, and hemorrhagic transformation in thrombolyzed patients with ischemic strokes [14,16,17]. Previous evidence has revealed that the calcium-binding protein S100β is characterized as a plasma biomarker of blood–brain barrier dysfunction, and disturbance of the blood–brain barrier is now shown to be the main initial feature in the pathogenesis of SVD [21]. To the best of our knowledge, astrocytes can release S100β by triggering stress, which promotes the proliferation of vascular smooth muscle cells by binding to receptors for advanced glycation end products (RAGE), a transmembrane receptor of the Ig super family in various cell types, thus resulting in narrowed blood vessel lumens [18]. Moreover, the activation of RAGE by S100β can stimulate the release of various inflammatory cytokines, the process of which plays an important role in cerebral microbleeds and is associated with SVD [22]. Gao et al*.* [18] indicates that the plasma levels of S100β are elevated in patients with SVD and signals the implication of this protein in the pathogenesis of SVD, which is largely consistent with our findings. Several studies have confirmed increased S100β in SVD, however, there are few previous studies further indicating the association of increased S100β levels and SVD with VCIND [23,24].

Our study also indicates that the MMSE and MoCA grades in the VCIND group are significantly lower than those in the NCI group and the normal control group. MMSE and MoCA are the two most common scales used to test cognitive impairment in various neurological dysfunctions, such as stroke and dementia [25,26]. MMSE was developed to assess cognitive dysfunction in Alzheimer’s disease, which is marked by difficulties in memory progress and language, and the scale is less frequently coupled with executive function, word-finding, and visual spatial ability deficits in early diagnosis [27]. By contrast, MoCA evaluates additional cognitive functions that are also influenced in Parkinson’s disease, particularly visual-, spatial-, and fronto-striatal abilities [25]. The results of our study show that the MMSE subitems of recall and executive function, and the MoCA subitems of attention, delay memory, abstract, visual space, and executive function are decreased in SVD patients with VCIND compared with those in the NCI group and normal control group. Furthermore, our data reveal that S100β levels exhibit a negative correlation with the MMSE total grade and subitems, including executive function, orientation, and calculation, and the MoCA total grade and subitems, including delay memory, attention, visual space, executive function, and naming. Similar to our findings, Chaves et al*.* [28] reported that S100β levels exhibit a negative correlation with MMSE scores in patients with cognitive dysfunction. Additionally, S100β levels are demonstrated to be related to MoCA scores in patients with neurological diseases [18], which confirmed the result of the study. In addition, an increased S100β level could be seen in SVD patients with hypertension in comparison with those with normal blood pressure. S100β, a calcium-binding protein, is predominantly produced by active astrocytes with a nerve growth factor-like (NGF) effect [29]. Increased abundance of NGF could be seen in the vein wall in hypertension [30], which could be a probable mechanism. However, no previous study has shown the direct link between S100β and hypertension, therefore, further study is needed to confirm the result.

## Conclusion

In conclusion, our study supports that the serum S100β level is elevated in SVD patients with VCIND. The MMSE and MoCA evaluations demonstrate a strong correlation of increased S100β and reduced cognitive function in SVD patients with VCIND. Therefore, the serum S100β level may be a biochemical marker for cognitive impairment in SVD patients. As a potential monitoring tool for cognitive dysfunction, the S100β level provides a target for the early intervention of SVD with VCIND. However, due to the limitation of funds, we failed to conduct a further study to look into whether S100β with other markers could help to discriminate the potential mechanism by which release of S100β affects the pathogenesis of SVD with VCIND and to determine more factors that contribute to cognitive impairment in SVD.

## Abbreviations

AD8: ascertain Dementia 8-item Questionnarie
CI: confidence interval
HAMD: hamilton depression rating scale
HDL-C: high density lipoprotein-cholesterol
HRP: horseradish peroxidase
LDL-C: low density lipoprotein-cholesterol
MMSE: mini-mental state examination
MoCA: Montreal cognitive assessment
NCI: no cognitive impairment
NGF: nerve growth factor
OR: odds ratio
PVC: polyvinyl chloride
RAGE: receptors for advanced glycation end products
ROC: receiver operating characteristic
SVD: small vessel disease
TC: total cholesterol
TG: triglyceride
VCIND: vascular cognitive impairment no dementia
